# Retraction: Sensing risk, fearing uncertainty: systems science approach to change

**DOI:** 10.3389/fncom.2016.00019

**Published:** 2016-02-22

**Authors:** Frontiers in Neuroscience Editorial Office

Frontiers retracts the paper: “Sensing risk, fearing uncertainty: systems science approach to change” (doi: 10.3389/fncom.2014.00030). Following a formal complaint concerning the publication cited above, the Specialty Chief Editors of Frontiers in Computational Neuroscience conducted an assessment of the article, according to the Frontiers complaints protocol. The Specialty Chief Editors concluded that the publication should not have been accepted in its published form, as it does not meet the standards of editorial and scientific soundness for Frontiers in Computational Neuroscience. This assessment was conducted in consultation with the Handling Editor, Dr Tobias A Mattei, who agreed to this conclusion. The author agrees to the retraction, commenting that the article was inappropriate for the Journal and its audience.

